# Molecular and karyological data confirm that the enigmatic genus *Platypholis* from Bonin-Islands (SE Japan) is phylogenetically nested within *Orobanche* (Orobanchaceae)

**DOI:** 10.1007/s10265-016-0888-y

**Published:** 2016-12-21

**Authors:** Xi Li, Tae-Soo Jang, Eva M. Temsch, Hidetoshi Kato, Koji Takayama, Gerald M. Schneeweiss

**Affiliations:** 1 Department of Botany and Biodiversity Research, University of Vienna, Rennweg 14, 1030 Vienna, Austria; 2 Makino Herbarium, Tokyo Metropolitan University, 1-1 Minami-Ohsawa, Hachioji-shi, Tokyo 192-0397 Japan; 3 Museum of Natural and Environmental History, Shizuoka, 5762 Oya, Suruga-ku, Shizuoka-shi, Shizuoka 422-8017 Japan

**Keywords:** Bonin Islands, Chromosome number, Molecular phylogeny, *Orobanche*, Parasitic plant, *Platypholis*

## Abstract

Molecular phylogenetic studies have greatly improved our understanding of phylogenetic relationships of non-photosynthetic parasitic broomrapes (*Orobanche* and related genera, Orobanchaceae), but a few genera have remained unstudied. One of those is *Platypholis*, whose sole species, *Platypholis boninsimae*, is restricted to the Bonin-Islands (Ogasawara Islands) about 1000 km southeast of Japan. Based on overall morphological similarity, *Platypholis* has been merged with *Orobanche*, but this hypothesis has never been tested with molecular data. Employing maximum likelihood and Bayesian analyses on a family-wide data set (two plastid markers, *matK* and *rps2*, and three nuclear markers, ITS, *phyA* and *phyB*) as well as on an ITS data set focusing on *Orobanche* s. str., it is shown that *P. boninsimae* Maxim. is phylogenetically closely linked to or even nested within *Orobanche* s. str. This position is supported both by morphological evidence and by the newly obtained chromosome number of 2*n* = 38, which is characteristic for the genus *Orobanche* s. str.

**Electronic supplementary material:**

The online version of this article (doi:10.1007/s10265-016-0888-y) contains supplementary material, which is available to authorized users.

## Introduction

Orobanchaceae have become a model group for studying the evolution of parasitic flowering plants (Westwood et al. 2010), because the family includes the full range of nutritional modes (from nonparasitic via photosynthetic parasitic to non-photosynthetic parasitic) as well as a number of pest species parasitic on economically important crop plants (Heide-Jørgensen 2008). For a better understanding of the evolution of parasitism and associated changes, a sound phylogenetic framework is needed. Despite enormous progress with respect to elucidating phylogenetic relationships within Orobanchaceae (McNeal et al. 2013), a considerable number of genera have not been studied yet using molecular phylogenetic approaches (Schneeweiss 2013), rendering our knowledge on phylogenetic relationships in Orobanchaceae incomplete.

**Fig. 1:**
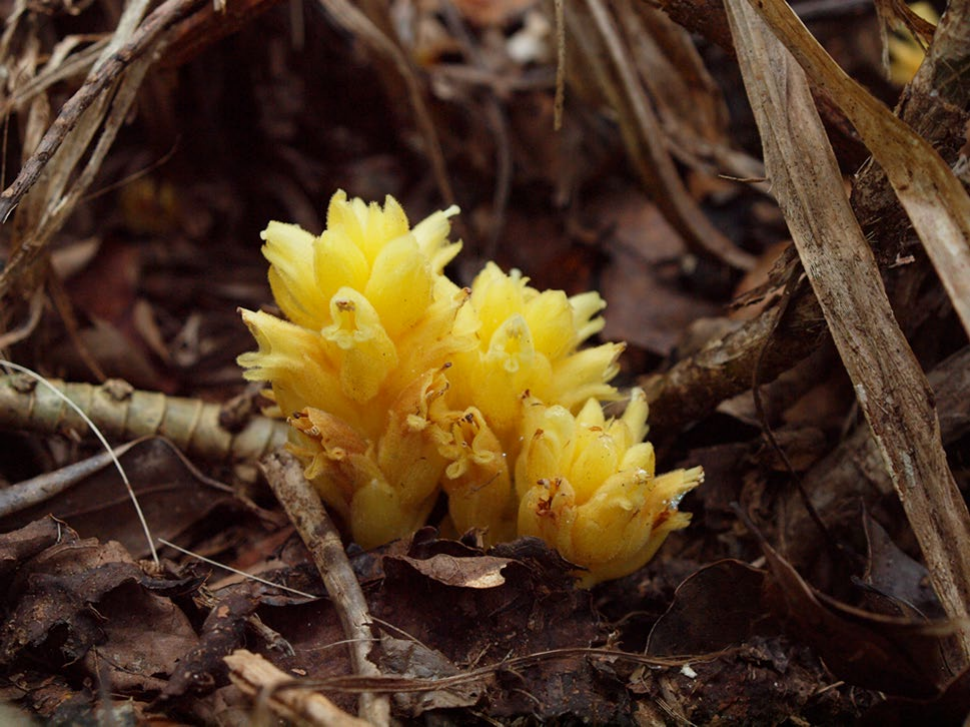
Habit of *Orobanche boninsimae* (syn. *Platypholis b*.) on Mt. Chibusayama, Hahajima Island (photo by H. Kato)

The highest diversity of non-photosynthetic parasitic (i.e., holoparasitic) species within Orobanchaceae is found in the exclusively holoparasitic *Orobanche* clade. While relationships and circumscription of its constituent genera are largely established (Schneeweiss 2013), molecular data are still lacking for the two East Asian genera *Phacellanthus* Siebold and Zucc. and *Platypholis* Maxim., the latter the focus of the present study. *Platypholis* contains a single species, *P. boninsimae* Maxim. (Fig. 1). It is endemic to the Bonin-Islands (Ogasawara I.) about 1000 km southeast of Japan, where it grows in shady, moist forests parasitizing mainly *Callicarpa subpubescens* Hook. and Arn. (Tuyama 1937). *Platypholis* was first described by Maximowicz (1886). He contrasted *Platypholis* with *Conopholis* Wallr., *Boschniakia* C.A.Mey. ex Bong., and *Lathraea* L. (the last not belonging to the *Orobanche* clade: McNeal et al. 2013; Schneeweiss 2013) that differ from *Platypholis* by non-exserted stamens as well as calyx and/or ovary structure. In conflict with Maximowicz’s (1886) description, Beck-Manngetta (1890, 1895, 1930) considered *Platypholis* to have three carpels with six placentas (instead of two carpels with four placentas) and consequently put it, together with *Xylanche* Beck (now merged with *Boschniakia* s. str.: Schneeweiss 2013) and *Phacellanthus*, into his Orobanchaceae tricarpellatae. Tuyama (1937) confirmed the observations of Maximowicz (1886) concerning the ovary structure of *Platypholis*. Furthermore, he considered *Platypholis* to be morphologically sufficiently similar to *Orobanche* s. l. to actually merge both genera and treat *P. boninsimae* as *Orobanche boninsimae* (Maxim.) Tuyama (Tuyama 1946). None of these hypotheses has, however, been tested with molecular data yet.

Here we want to clarify the phylogenetic position of *Platypholis* by testing previous hypotheses with respect to the phylogenetic position of *Platypholis* as distinct from *Orobanche* L. (Beck-Mannagetta 1890, 1895, 1930; Maximowicz 1886; Zhang 1988) versus within *Orobanche* (Tuyama 1937, 1946). To this end, we conducted phylogenetic analyses on a family-wide data set (comprising two plastid markers, *matK* and *rps2*, and three nuclear markers, ITS, *phyA* and *phyB*) as well as on an ITS data set focusing on *Orobanche* s. str. (see Schneeweiss 2013, for details on this narrower circumscription of *Orobanche*). As chromosome numbers and genome size data have been found to be phylogenetically informative in the *Orobanche* clade in general and in *Orobanche* s. l. in particular (Schneeweiss et al. 2004b; Weiss-Schneeweiss et al. 2006), these data were obtained as well. Specifically, if *Platypholis* indeed belongs to *Orobanche* (Tuyama 1937, 1946) we expect *Platypholis* to have a chromosome base number of *x* = 19.

## Materials and methods

### Plant material

Material of *Platypholis* was collected in 2014 in Higashidaira, Chichijima Island, Ogasawara (Bonin) Islands, Japan; the voucher is deposited at WU. For karyological and cytological investigation, young flower buds were fixed in the field in 3:1 ethanol:glacial acetic acid for at least 24 h at room temperature and stored at −20 °C until further use.

### DNA extraction, PCR and sequencing

Total DNA was extracted using the DNeasy Plant Mini Kit (Qiagen, Hilden, Germany) following the manufacturer’s instructions. Two plastid loci (*matK, rps2*) as well as three nuclear loci (ITS, *phyA* and *phyB*) that have been successfully used in previous phylogenetic studies of Orobanchaceae (McNeal et al. 2013) were amplified using primers listed in Table 1; new or modified primers were designed by eye from available alignments. Amplification of the plastid markers and of ITS was done in a volume of 15.7 µL containing 7 µL KAPA2G Fast2x ReadyMix (Peqlab, Vienna, Austria), 0.5 µL each of 10 µM primer, 1 µL of DNA extract of unknown concentration, and 7 µL sterile water. Amplification of the two phytochrome regions was done in a volume of 15.5 µL containing 0.375 U of Platinum High Fidelity Taq (Invitrogen, Carlsbad, California), 1.5 µL of 10× PCR buffer, 0.8 µL of 50 mM MgSO_4_, 0.15 µL of 10 mM dNTPs, 0.5 µL each of 10 µM primer, 0.7 µL of DNA extract of unknown concentration and 10.85 µL sterile water. PCR conditions for ITS amplification were: denaturation for 4 min at 94 °C; 35 cycles each with 30 s at 94 °C, 30 s at 48 °C, 1 min at 72 °C; and final elongation for 10 min at 72 °C. For the remaining four loci (*rps2, matK, phyA* and *phyB*) a touchdown PCR protocol was used, thus obviating potential problems due to degenerate primers. The PCR conditions were: 2 min at 94 °C; 9 cycles each with 30 s at 94 °C, 15 s at 67 °C (decreasing the annealing temperature by 1 °C at each subsequent cycle, so that in the 9th cycle the annealing temperature was 59 °C), 90 s at 70 °C; 21 cycles each with 30 s at 94 °C, 30 s at 57 °C, 90 s at 70 °C; 12 cycles with 30 s at 94 °C, 45 s at 62 °C, 90 s at 70 °C; a final elongation for 7 min at 70 °C. PCR products were purified using Exonuclease I and FastAP thermosensitive alkaline phosphatase (Fisher Scientific, St. Leon-Rot, Germany) following the manufacturer’s instructions. Cycle sequencing reactions were performed using 5 µL of purified template, 1 µL of primer (3.2 µM) and 1 µL BigDye Terminator (Applied Biosystems, Foster City, California), cleaned with Sephadex G-50 Fine (GE Healthcare Bio-Sciences, Uppsala, Sweden) and sequenced on an ABI 3730 DNA Analyzer capillary sequencer (Applied Biosystems).

**Table 1:** Amplification primers

Primer		References
Name	Sequence (5′–3′)	
*matK*		
trnK 3914F di	GGGGTTGCTAACTCAACGG	Johnson and Soltis (1995)
matK550Fca	TGGAAATCTTGGTTCAAACTCTTCG	This study
matK-50Fdi	GTTTTGACTGTATCGCACTATGTATC	Demaio et al. (2011)
matK 950r	CCACARCGAAAAATRMCATTGCC	Young et al. (1999)
matK 1349r	CTTTTGTGTTTCCGAGCYAAAGTTC	Young et al. (1999)
trnK-R2*	CTCGAACCCGGAACTAGTCGG	Castello et al. (2016)
*rps2*		
rps2-47F	CTCGTTTTTTATCTGAAGCCTG	dePamphilis et al. (1997)
rps2-58F	AAATGGAATCCTAAAATGGCA	This study
rps2-661R	ACCCTCACAAATAGCGAATACCAA	dePamphilis et al. (1997)
ITS		
ITS AB101	ACGAATTCATGGTCCCGTGAAGTGTTCG	Schneeweiss et al. (2004a)
ITS AB102	TAGAATTCCCCGGTTCGCTCGCCGTTAC	Schneeweiss et al. (2004a)
*phyA*		
PHYA230f	GACTTTGARCCNGTBAAGCCTTAYG	Mathews and Donoghue (1999)
PHYA_Newa678r	GTCTCRATCARACGAACCATCTC	This study
*phyB*		
PHYB7f	CACAGGATAGAYGTRGGRGT	This study
NewPHYB_b678r.oro	GTCTCTATCAACCTAAYCATCTC	This study (modified from McNeal et al. 2013)

### Phylogenetic analyses

Sequences were assembled and edited using SeqMan II 5.05 (DNAStar Inc., Madison, USA). The newly obtained data of *Platypholis* were added and aligned by eye to the existing single and combined marker alignments of McNeal et al. (2013), available from TreeBase (http://treebase.org) under study number 13942, using BioEdit 7.2.1 (Hall 1999). Likewise, ITS sequences of *Platypholis* were added to the alignment of Frajman et al. (2013) that focuses on *Orobanche* s. str. and consequently has a much denser sampling within that genus. Sequence alignments are available from ResearchGate under doi:10.13140/RG.2.1.4124.1203.

The best-fit substitution models were identified using the Akaike Information Criterion (AIC) and the Bayesian Information Criterion (BIC) as implemented in jModelTest 2.1.6 (Darriba et al. 2012). We tested 44 substitution models (11 substitution schemes, allowing unequal frequencies and/or rate heterogeneity across sites modeled by a gamma distribution, but no proportion of invariable sites due to identifiability issues: Yang 2014) on maximum-likelihood (ML) optimized topologies obtained following SPR (Subtree Pruning and Regrafting) branch swapping. For each dataset, the General Time Reversible (GTR) model (Tavaré 1986) including rate heterogeneity across sites described by a gamma distribution was selected. Maximum likelihood analyses were conducted using RAxML 8.1 (Stamatakis et al. 2014) employing the fast bootstrap approach (Stamatakis et al. 2008) with 1000 bootstrap replicates. Bayesian inference was done using MrBayes 3.2.3 (Ronquist et al. 2012). Values for all parameters, such as the shape of the gamma distribution (approximated using six discrete rate categories) or the substitution rates, were estimated during the analysis. For partitioned analyses (combined data set only), partitions were allowed to evolve under different rates (ratepr = variable). Four Monte Carlo Markov (MCMC) chains were run simultaneously starting from different random starting trees for 20 million generations, with trees sampled every 5000th generation. After combining 3600 trees from each run (i.e., after discarding 10% of samples as burn-in, when the MCMC chain had reached stationarity as confirmed by visual inspection of traces and standard deviations of split variances being below 0.01), posterior probabilities were estimated from these 14,400 posterior trees and were plotted on a majority rule consensus tree.

### Chromosome number and genome size

The chromosome number of *Platypholis* was determined from meiotic divisions in pollen mother cells (PMCs) and from first mitosis in developing microspores using the standard Feulgen staining technique (Schneeweiss et al. 2004b). Fixed material was hydrolyzed in 5N HCl for 30 min at room temperature, washed with tap water and stained with Schiff’s reagent (Merck, Darmstadt, Germany) in darkness for 1 h (Jang et al. 2013). Chromosome spreads were prepared by squashing stained anthers in a drop of acetic acid (60%) under the cover-slip, and analyzed using an AxioImager M2 microscope (Carl Zeiss, Vienna, Austria). Preparations with a minimum of 15 good quality chromosome spreads were analyzed. Images were acquired with a CCD camera and files processed using AxioVision 4.8 (Carl Zeiss). The karyotype was made from these images in PhotoPaint X7 (Corel Corp., Ottawa, Ontario).

Fixed flower buds were transferred to ethanol and stored in the deep freezer. For preparation for genome size estimation, plant tissue was rehydrated and hydrolyzed for 60 min in 5N HCl at 20 °C (Greilhuber and Temsch 2001) together with root tips from the internal standard (*Pisum sativum* L. ‘Kleine Rheinländerin’, 1C = 4.42 pg: Greilhuber and Ebert 1994). After washing with water, the samples were stained with Schiff´s reagent over-night in the refrigerator. The dye was removed by washing six times with SO_2_-water over a total period of 45 min. Subsequently, the stained tissue was squashed on slides, frozen, and after removal of cover slips fixed in 96% ethanol, dried and stored until measurement. Measurements of the Integrated Optical Densities (IOD) were conducted on the Cell Image Retrieval and Evaluation System (CIRES, Kontron, Munich), which was equipped with a CCD DXC 390P camera (Sony, Tokyo, Japan) and an Axioscope microscope (Carl Zeiss). From each slide, for both the object and the internal standard, 10 prophase and 10 telophase nuclei were measured. Per slide a 1C-value was calculated using the formula (mean IOD_Obj_/mean IOD_Std_)*1C-value_Std_.

## Results

**Fig. 2:**
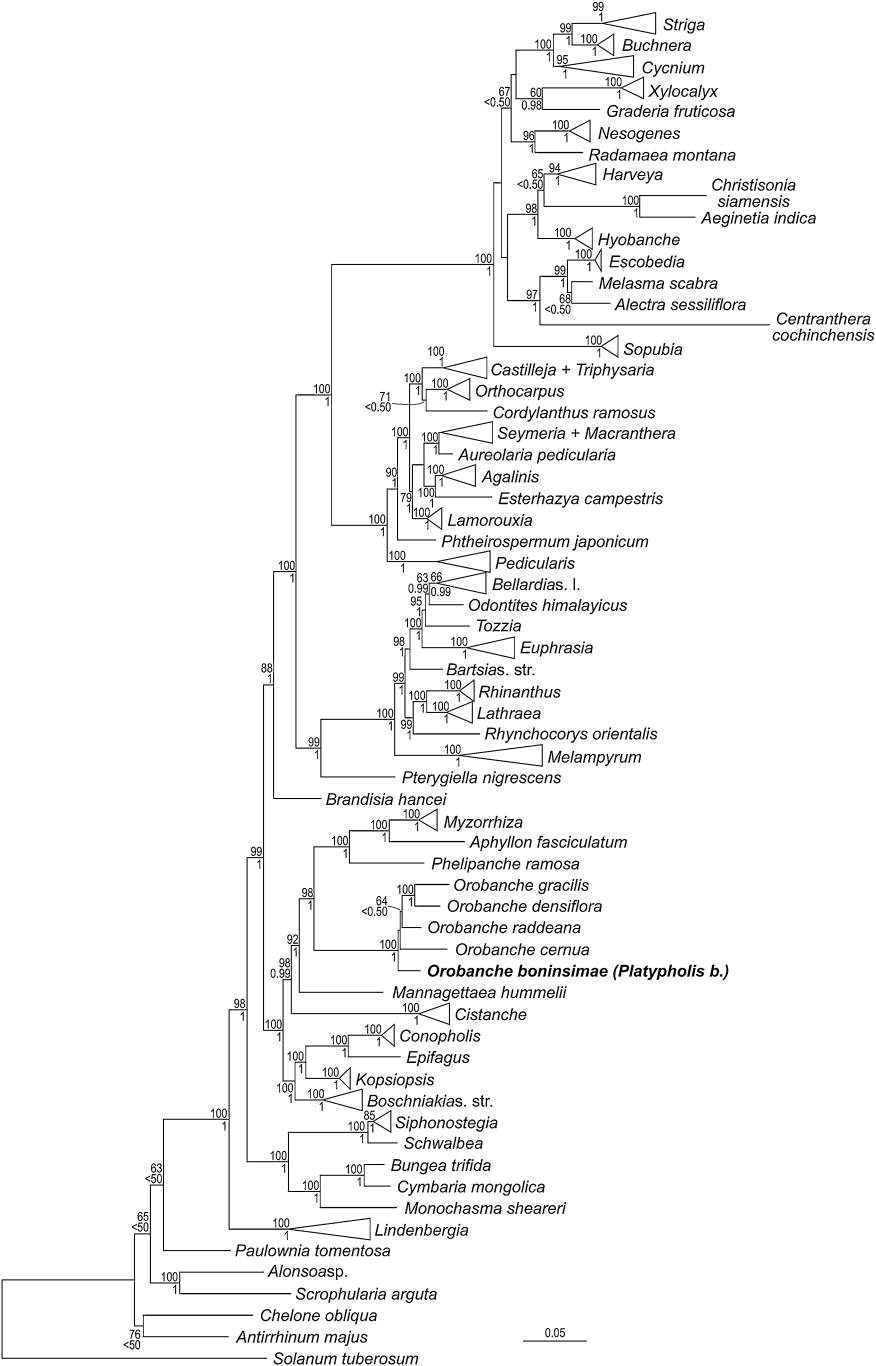
Phylogenetic placement of *Orobanche boninsimae* (syn. *Platypholis b*.) within Orobanchaceae inferred using maximum likelihood on a five marker combined data set (*matK*, rps2, ITS, *phyA, phyB*). *Numbers* at branches are maximum likelihood bootstrap support values (60 or higher) and posterior probabilities (0.5 or higher). *Solanum tuberosum* L. was chosen as outgroup

**Fig. 3:**
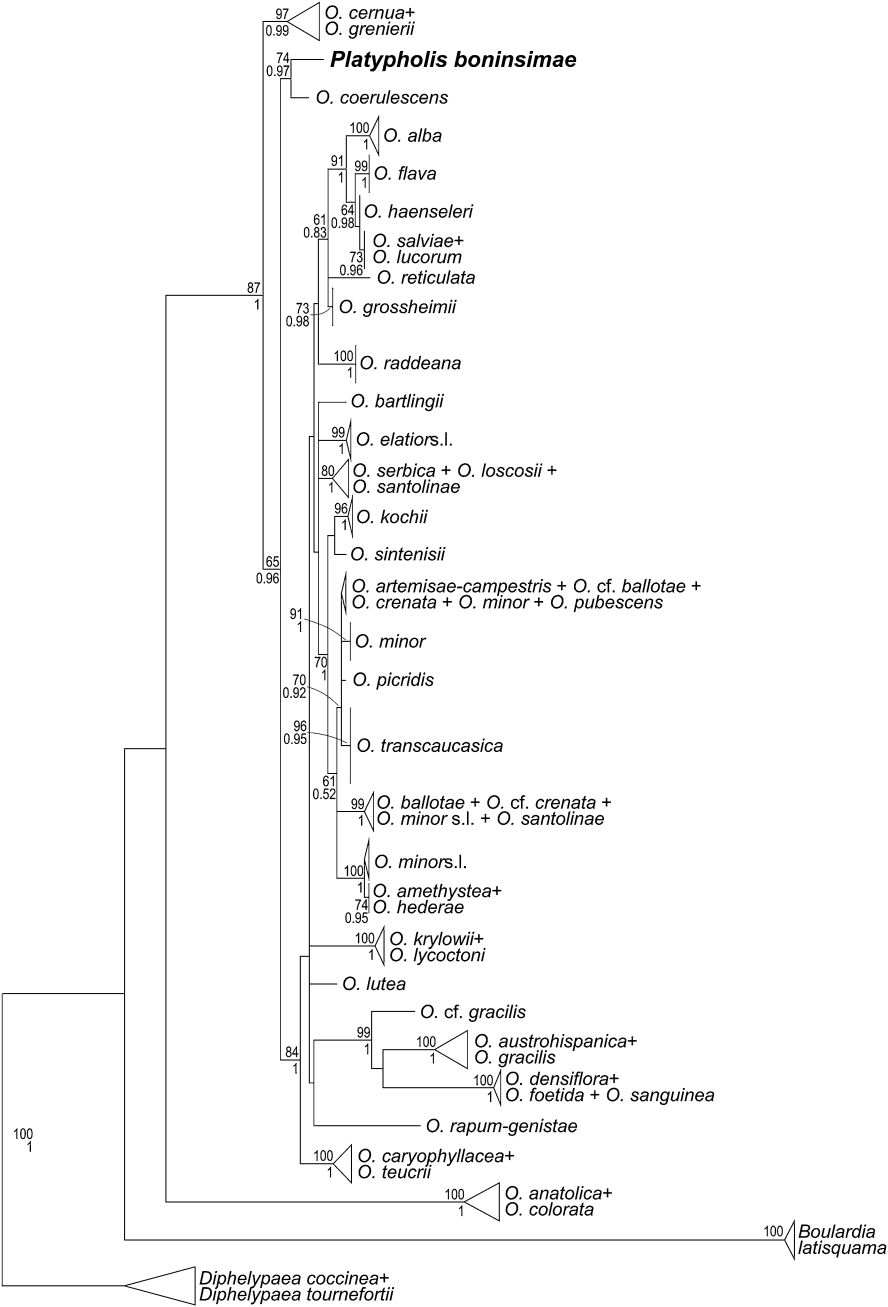
Phylogenetic placement of *Orobanche boninsimae* (syn. *Platypholis b*.) within *Orobanche* s. str. (i.e., also excluding *Boulardia*: Schneeweiss 2013) inferred using maximum likelihood on an ITS data set. *Numbers* at branches are maximum likelihood bootstrap support values (60 or higher) and posterior probabilities (0.5 or higher). *Diphelypaea* Nicolson was chosen as outgroup (Schneeweiss et al. 2004a)

Newly obtained sequences of *matK, rps2*, ITS, *phyA* and *phyB* are available from GenBank under accession numbers KU647699, KU647702, KU647698, KU647700 and KU647701, respectively. Phylogenetic analyses of the family-wide data sets (single marker and concatenated data sets) congruently place *Platypholis* within the *Orobanche* clade as sister to or nested within *Orobanche* s. str. (BS = 100, PP = 1; Online Resource 1; Fig. 2), but low resolution and/or insufficient support (except for ITS: Fig. 3; Fig. S3 in Online Resource 1) prevent the precise phylogenetic position of *Platypholis* being identified (Fig. 2; Online Resource 1). Although phylogenetic relationships among lineages inferred from single markers are not fully congruent (Online Resource 1), well-supported incongruences involving *Platypholis* are lacking and those involving the remaining taxa have been found to be not statistically significant (McNeal et al. 2013). Analyses of a data set focusing on *Orobanche* s. str. place *Platypholis* firmly within *Orobanche* s. str. (BS = 87, PP = 1.00, Fig. 3), where it is inferred as sister species to *O. coerulescens* Stephan (BS = 74, PP = 0.97, Fig. 3).

**Fig. 4:**
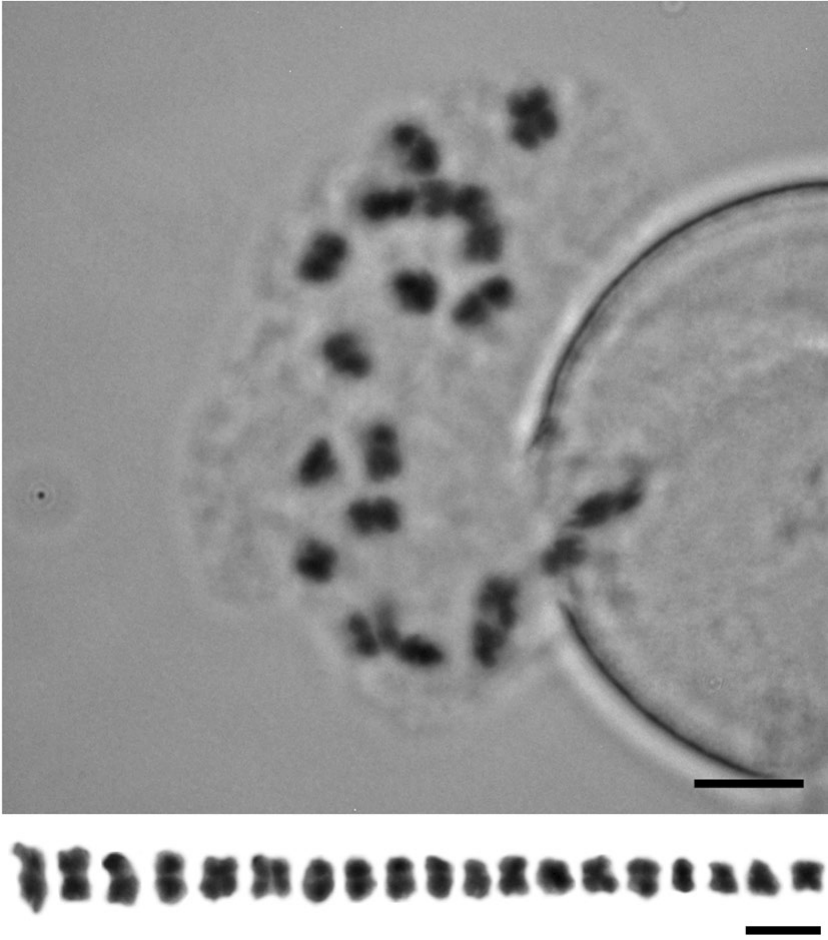
Chromosomes and karyotype of *Orobanche boninsimae* (syn. *Platypholis b*.): *n* = 19 (metaphase of first mitotic division in microspore). *Scale bar* 5 μm

*Platypholis* is diploid with a chromosome number of 2*n* = 2*x* = 38 (Fig. 4). All chromosomes are metacentric to submetacentric and their lengths range from 2 to 5 µM (Fig. 4), resulting in a Haploid Karyotype Length (HKL) of 54.83 µM. The nuclear DNA amount (1C) of *Platypholis*, calculated as average from four slide pairs, is 7.28 pg (S.D. 0.1805, C.V. 2.48%).

## Discussion

The monotypic genus *Platypholis* has not been included in any molecular phylogenetic study of Orobanchaceae to date and its precise placement within the family remained uncertain (Schneeweiss 2013). Using data from two plastid and three nuclear loci, it is shown that *Platypholis* phylogenetically belongs to the *Orobanche* clade (Fig. 2, Online Resource 1) and is sister to or even nested within *Orobanche* s. str. (Fig. 3). The uncertainty concerning the precise placement of *Platypholis* may be due to issues of paralogy in nuclear markers, especially ITS (Álvarez and Wendel 2003). As neither gel visualization of PCR products nor direct sequencing indicated any presence of paralogues and as there are no strongly supported, but rather contradicting phylogenetic relationships inferred from plastid versus nuclear markers (Online Resource 1), we consider it unlikely that our inferences are misled by paralogues. Alternatively, incongruences between different markers might be due to incomplete lineage sorting, which can be accommodated by using species tree estimation methods. This will, however, require much larger data sets, especially if a possible negative affect of missing data is to be avoided (Xi et al. 2016), which goes beyond the scope of this study.

A close relationship of *Platypholis* and *Orobanche* s. str. is also supported by the shared chromosome number of 2*n* = 38 (Fig. 4; Schneeweiss et al. 2004b). Hence, both molecular phylogenetic and karyological data refute (implicit) hypotheses of Maximowicz (1886) and Beck-Mannagetta (1890, 1895, 1930) on a closer relationship to *Lathraea, Conopholis*, and/or *Boschniakia* (no data available for *Phacellanthus*) and instead support Tuyama (1937, 1946), who suggested a close relationship to *Orobanche*. Tuyama (1937) also noted several morphological characters that *Platypholis* shares with all or at least some species of *Orobanche* s. str., including the absence of bracteoles, the flowers being sessile, the calyx being divided into two lateral sepals, the basal insertion of stamens, and the ovary structure (two-carpellate ovaries with four placentae). The last is of particular relevance, because Beck-Mannagetta (1890, 1895, 1930) placed *Platypholis* in his Orobanchaceae tricarpellatae based on the perceived presence of three carpels and six separate placentae (see Fig. 24G in Beck-Mannagetta 1930: 331), while he classified *Orobanche* s. str. (as *O*. sect. *Ospreolon*) within his Orobanchaceae bicarpellatae, due to the presence of two carpels and four separate placentae. Beck’s observations are even more puzzling, because Maximowicz (1886), when describing *Platypholis*, had already indicated the presence of four placentae only, which Beck dutifully reported, albeit with reservations (Beck-Mannagetta 1930: 332 “sec. Maximowicz solum 4”: “according to Maximowicz only 4”). Taxonomically, the genus *Platypholis* can no longer be upheld and, following Tuyama (1937, 1946) and subsequent Japanese authors, its single species is to be treated as member of *Orobanche* s. str. as *O. boninsimae*.

The phylogenetic placement of *O. boninsimae* within *Orobanche* s. str. is less certain. A closer relationship to *O. coerulescens*, as suggested by ITS data (Fig. 3), is supported by geographic proximity, as *O. coerulescens* (lacking from the Bonin Islands) is the sole *Orobanche* species occurring on the main islands of Japan (Shimane). *Orobanche boninsimae* differs from *O. coerulescens* and other *Orobanche* species by having exserted stamens (unique within the genus; Fig. 1), a stem that is strongly branched at the caudex, larger chromosomes (2–5 µM vs. 1–3 µM: Schneeweiss et al. 2004b) and a correspondingly larger genome (7.28 pg/1C vs. 1.45–5.83 pg/1C: Weiss-Schneeweiss et al. 2006). The considerably larger genome observed in *O. boninsimae* may be connected to life-history (the species is perennial: Tuyama 1937; reports by Abe (2006) that *O. boninsimae* is annual are incorrect) or changes in breeding system, as noted in other plant groups (Albach and Greilhuber 2004; Price et al. 2005).

## Electronic supplementary material

Below is the link to the electronic supplementary material.

Supplementary material 1 (PDF 762 KB)
