# Supplementary material for: Molecular and karyological data confirm that the enigmatic genus Platypholis from Bonin-Islands (SE Japan) is phylogenetically nested within Orobanche (Orobanchaceae)
Source: J Plant Res. 2016 Dec 21;130(2):273–80. doi: 10.1007/s10265-016-0888-y (PMC5318490; doi:10.1007/s10265-016-0888-y)
Supplement: Supplementary file 1 — Supplementary material 1 (PDF 762 KB) [file 10265_2016_888_MOESM1_ESM.pdf]

Title: Molecular and karyological data confirm that the enigmatic genus *Platypholis* from Bonin-Islands (SE Japan) is phylogenetically nested within *Orobanche* (Orobanchaceae)

Journal: Journal of Plant Research

Authors: Xi Li, Tae-Soo Jang, Eva M. Temsch, Hidetoshi Kato, Koji Takayma, Gerald M. Schneeweiss

Corresponding author: Gerald M. Schneeweiss, Department of Botany and Biodiversity, University of Vienna, Rennweg 14, A-1030 Vienna, Austria. Fax: +43 1 4277 9541.E–mail: gerald.schneeweiss@univie.ac.at

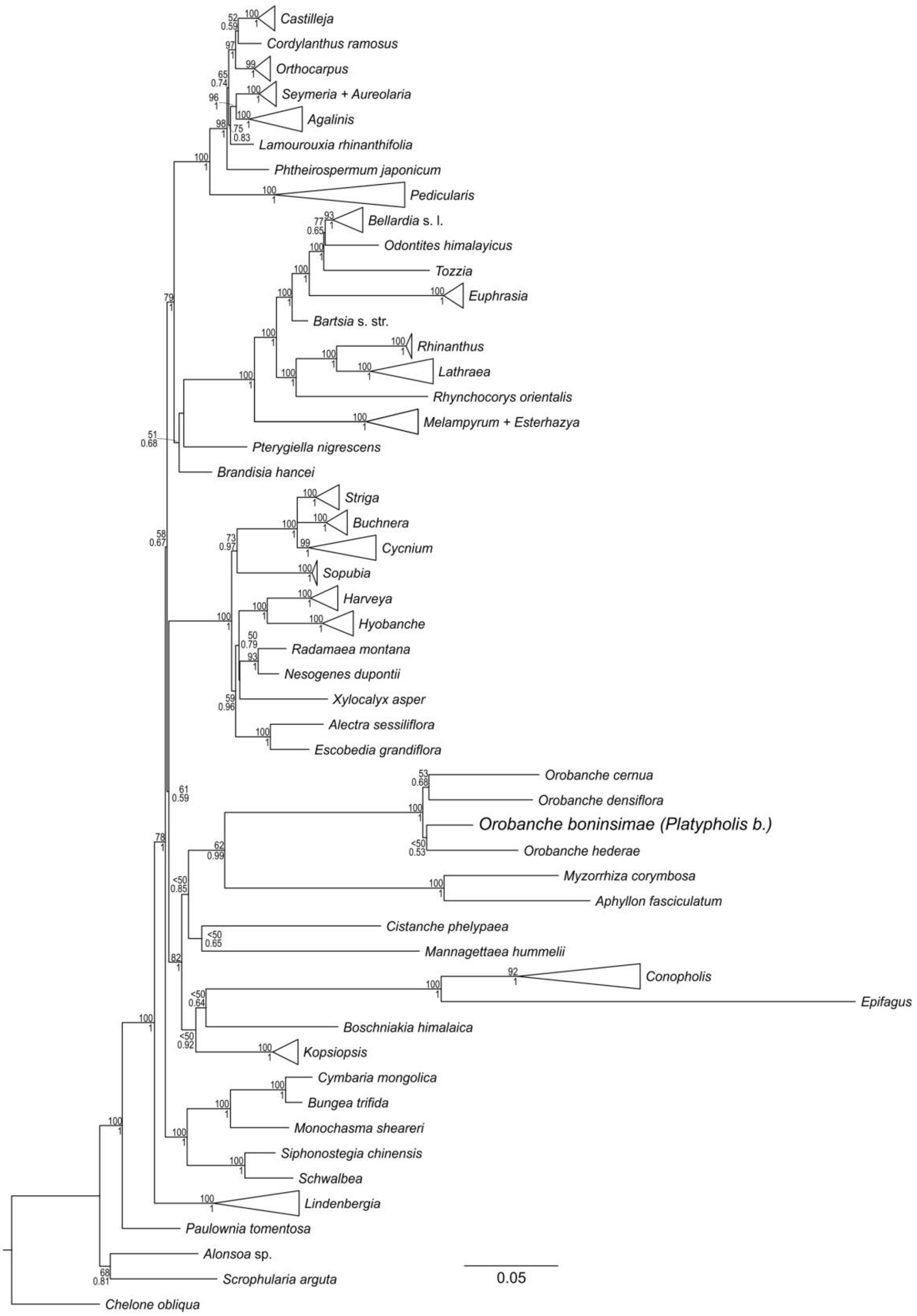

Fig. S1 Phylogenetic placement of *Orobanche boninsimae* (syn. *Platypholis b.*) within Orobanchaceae inferred using maximum likelihood on a *matK* data set. Numbers at branches are maximum likelihood bootstrap support values (50 or higher) and posterior probabilities (0.5 or higher).

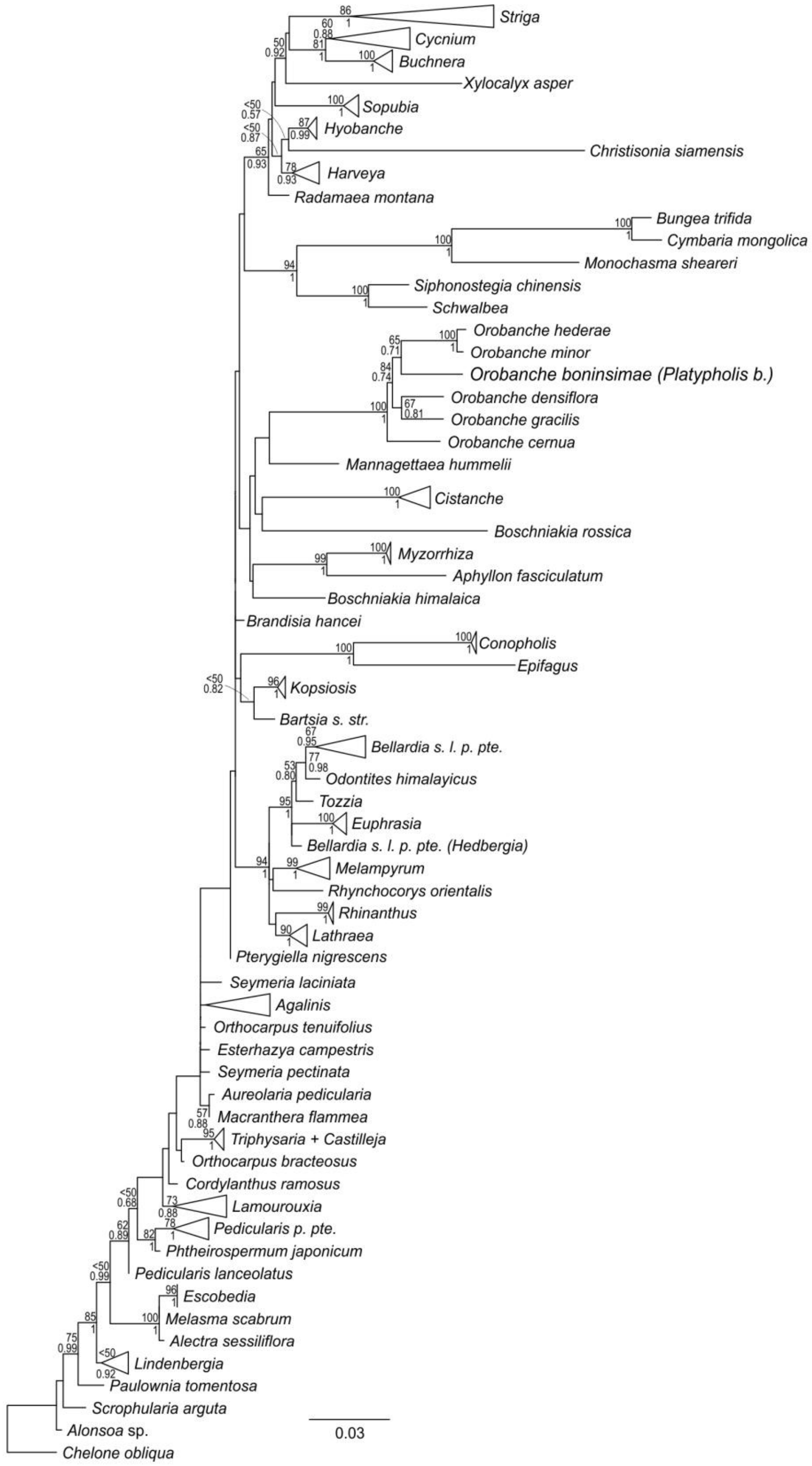

Fig. S2 Phylogenetic placement of *Orobanche boninsimae* (syn. *Platypholis b.*) within Orobanchaceae inferred using maximum likelihood on a *rps2K* data set. Numbers at branches are maximum likelihood bootstrap support values (50 or higher) and posterior probabilities (0.5 or higher).

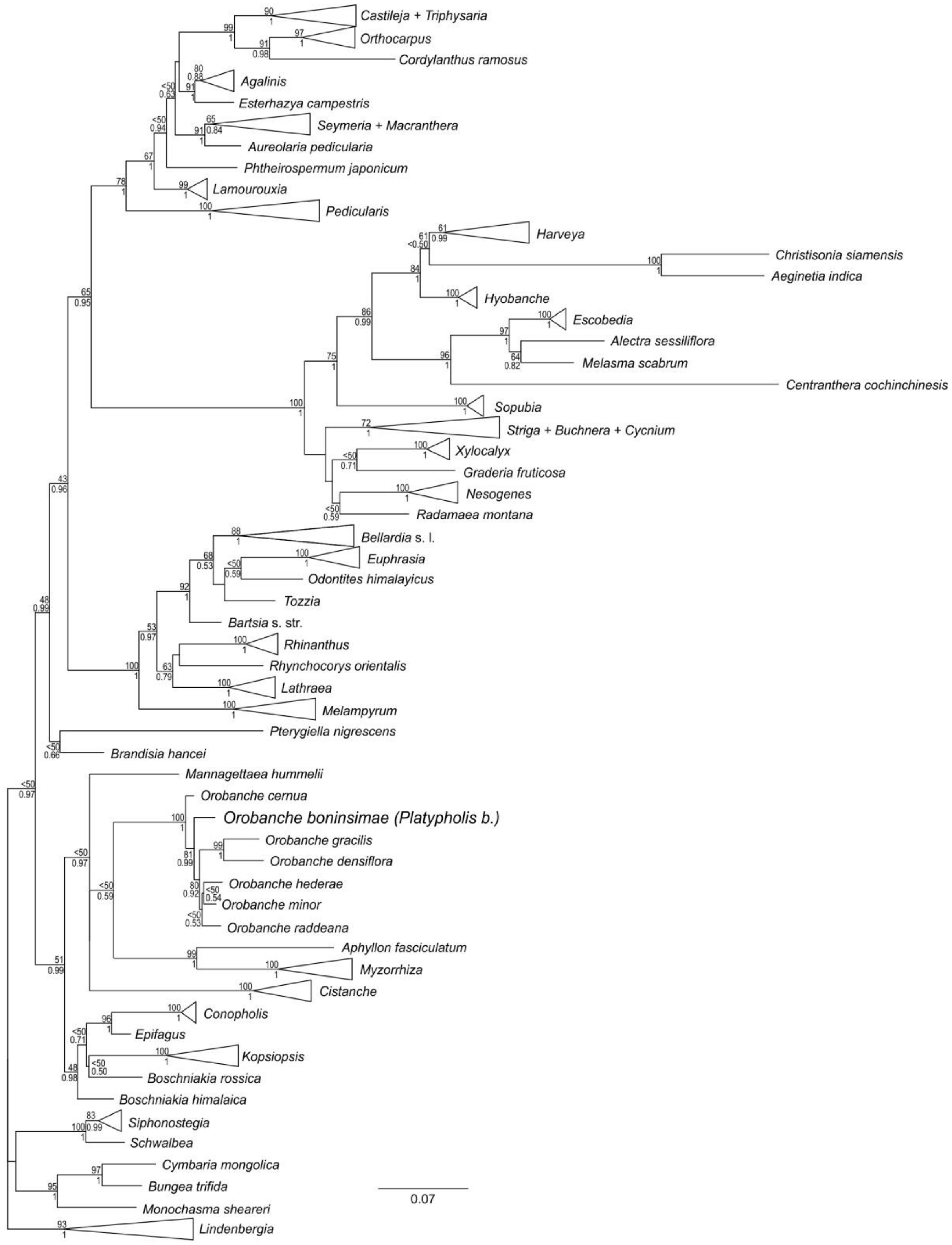

Fig. S3 Phylogenetic placement of *Orobanche boninsimae* (syn. *Platypholis b.*) within Orobanchaceae inferred using maximum likelihood on a ITS data set. Numbers at branches are maximum likelihood bootstrap support values (50 or higher) and posterior probabilities (0.5 or higher).

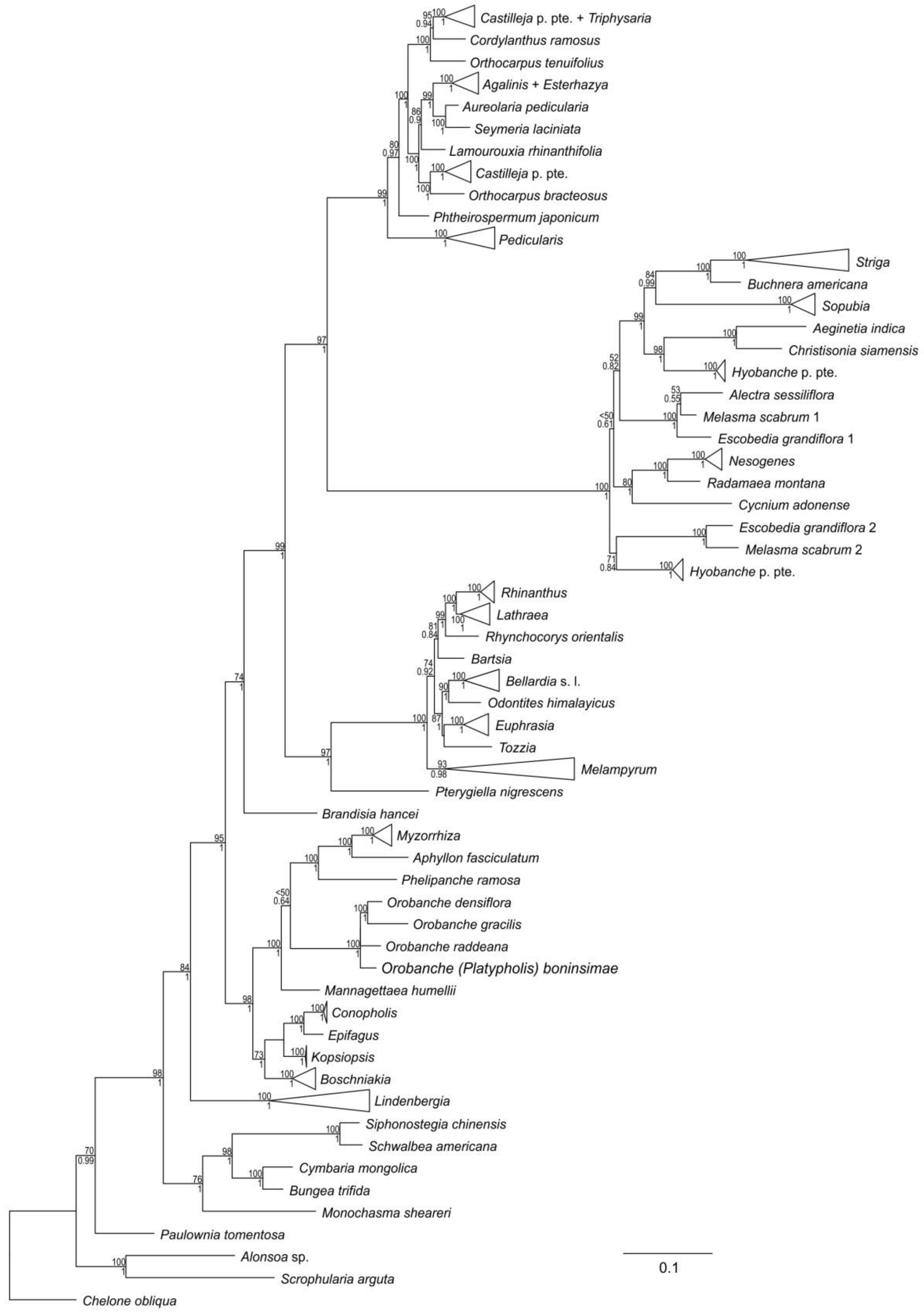

Fig. S4 Phylogenetic placement of *Orobanche boninsimae* (syn. *Platypholis b.*) within Orobanchaceae inferred using maximum likelihood on a *phya* data set. Numbers at branches are maximum likelihood bootstrap support values (50 or higher) and posterior probabilities (0.5 or higher).

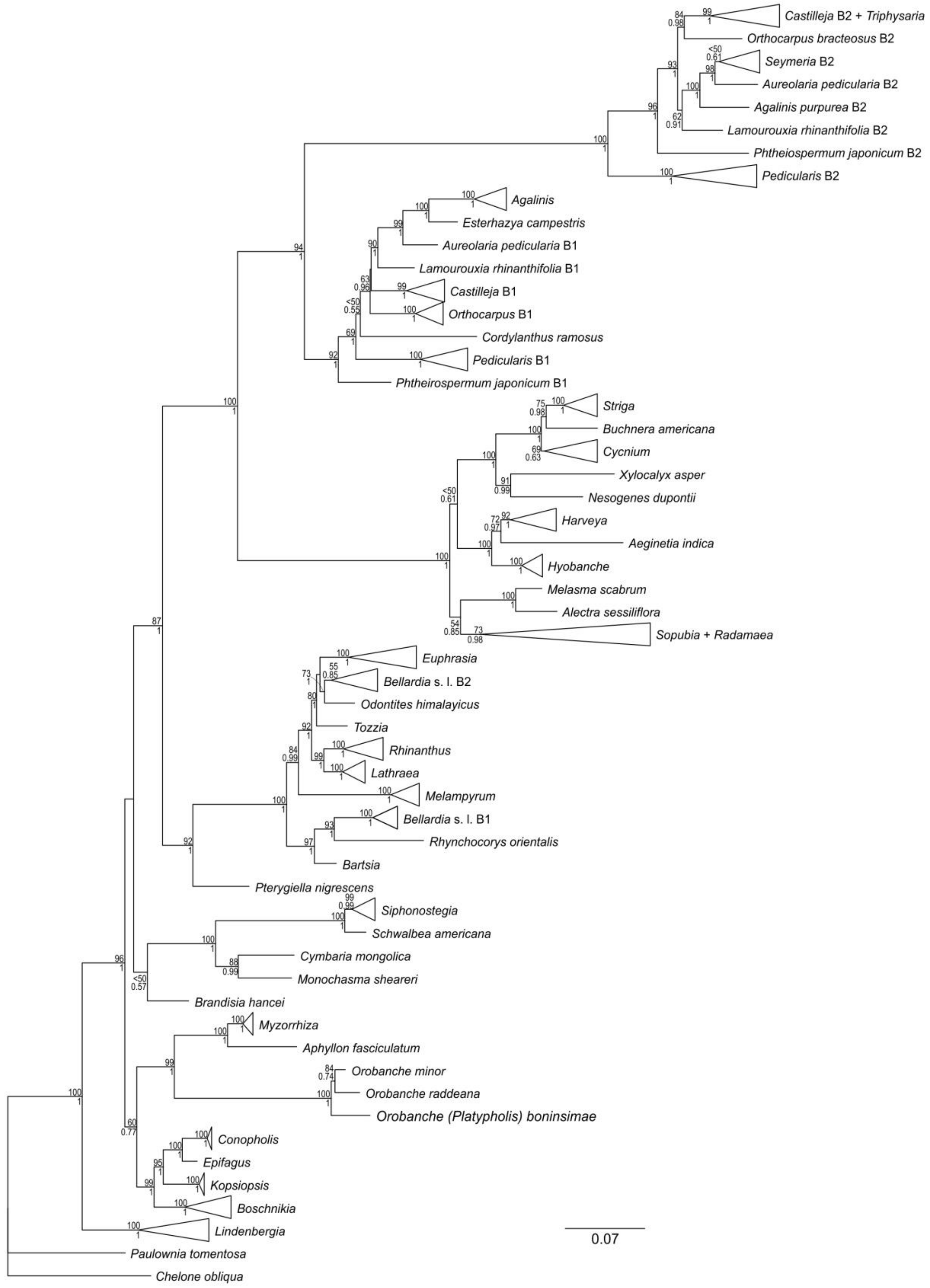

Fig. S5 Phylogenetic placement of *Orobanche boninsimae* (syn. *Platypholis b.*) within Orobanchaceae inferred using maximum likelihood on a *phyb* data set. Numbers at branches are maximum likelihood bootstrap support values (50 or higher) and posterior probabilities (0.5 or higher).
